# Development and implementation of a transmural palliative care consultation service: a multiple case study in the Netherlands

**DOI:** 10.1186/s12904-021-00767-6

**Published:** 2021-06-05

**Authors:** Marijanne Engel, Arianne Stoppelenburg, Andrée van der Ark, Floor M. Bols, Johannis Bruggeman, Ellen C.J. Janssens-van Vliet, Johanna H. Kleingeld-van der Windt, Ingrid E. Pladdet, Angelique E.M.J. To-Baert, Lia van Zuylen, Agnes van der Heide

**Affiliations:** 1 Department of Public Health, Erasmus MC, University Medical Center Rotterdam, Dr. Molewaterplein 40, 3015 GD Rotterdam, The Netherlands; 2 Center of Expertise in Palliative Care, Leiden University Medical Center, Leiden, The Netherlands; 3 Department of Palliative Care, Elisabeth-TweeSteden Hospital, Tilburg, The Netherlands; 4 General practice Bruggeman, Zwijndrecht, The Netherlands; 5 Department of Medical Oncology, Admiraal de Ruyter Hospital (Adrz), Goes, The Netherlands; 6 Palliative Care Network Waardenland, Dordrecht, The Netherlands; 7 SVRZ Ter Valcke, Goes, The Netherlands; 8 Department of Medical Oncology, Amphia Hospital, Breda, The Netherlands; 9 Department of Medical Oncology, Amsterdam University Medical Centers, Amsterdam, The Netherlands

**Keywords:** Palliative care, Continuity of patient care, Integrated care, Hospitals, Home care services, Generalist palliative care, Specialist palliative care, Transmural collaboration

## Abstract

**Background:**

In the Netherlands, healthcare professionals attending patients in the last phase of life, can consult an expert palliative care team (PCT) in case of complex problems. There are two types of PCTs: regional PCTs, which are mainly consulted by general practitioners, and hospital PCTs, which are mainly consulted by healthcare professionals in the hospital. Integration of these PCTs is expected to facilitate continuity of care for patients receiving care in different settings. We studied facilitators and barriers in the process of developing and implementing an integrated transmural palliative care consultation service.

**Methods:**

A multiple case study was performed in four palliative care networks in the southwest Netherlands. We aimed to develop an integrated transmural palliative care consultation service. Researchers were closely observing the process and participated in project team meetings. A within-case analysis was conducted for each network, using the Consolidated Framework for Implementation Research (CFIR). Subsequently, all findings were pooled.

**Results:**

In each network, project team members thought that the core goal of a transmural consultation service is improvement of continuity of palliative care for patients throughout their illness trajectory. It was nevertheless a challenge for hospital and non-hospital healthcare professionals to arrive at a shared view on goals, activities and working procedures of the transmural consultation service. All project teams experienced the lack of evidence-based guidance on how to organise the service as a barrier. The role of the management of the involved care organisations was sometimes perceived as unsupportive, and different financial reimbursement systems for hospital and out-of-hospital care made implementation of a transmural consultation service complex. Three networks managed to develop and implement a transmural service at some level, one network did not manage to do so.

**Conclusions:**

Healthcare professionals are motivated to collaborate in a transmural palliative care consultation service, because they believe it can contribute to high-quality palliative care. However, they need more shared views on goals and activities of a transmural consultation service, more guidance on organisational issues and appropriate financing. Further research is needed to provide evidence on benefits and costs of different models of integrated transmural palliative care consultation services.

## Background

Patients with a limited life-expectancy due to progressive illness or frailty often need care from different care providers in different care organisations [1]. The majority are at least once transferred between different care settings during the last months of life [2]. Adequate transmural care and collaboration between healthcare professionals from different organisations is therefore important [3–5]. Transmural care should be 'attuned to the needs of the patient and provided on the basis of co-operation and co-ordination between general and specialist caregivers, with shared overall responsibility and specification of delegated responsibilities' [6]. Transmural care typically involves collaboration between healthcare professionals working in and outside the hospital setting [7, 8]. During the last decade it has been increasingly recognised that the quality of collaboration between healthcare professionals from different care organisations is often not optimal [9–11].

In the Netherlands, one of the strategies to promote transmural collaboration in palliative care is the establishment of so-called regional palliative care networks by the Dutch Ministry of Health, Welfare and Sport [12, 13]. In these networks, care organisations such as hospitals, care and nursing homes, home care organisations, general practitioners, hospices and volunteers work together to optimise the provision of palliative care within a specific region. Networks have a basic structure that includes a steering group or management team consisting of representatives from participating care organisations, and a patient care-oriented member group consisting of healthcare professionals. Each palliative care network has appointed a network coordinator [14]. In total there are 65 palliative care networks, covering the whole country [14].

One of the main tasks of palliative care networks is to facilitate the integration of generalist palliative care, provided by physicians and nurses and other healthcare professionals in all care settings, and specialist palliative care, for more complex cases [15–17]. Within networks, generalist care providers attending patients with complex problems can consult a regional expert palliative care team (PCT). PCTs typically include palliative care specialists from several disciplines. They provide advice to the physician or nurse attending the patient, but do not take over care [18]. Regional PCTs can be consulted by healthcare professionals in all settings, but the majority of requests for advice come from general practitioners [19]. Hospital PCTs were originally intended to improve the quality of hospital palliative care and most hospital PCTs can only be consulted for inpatients [20]. In the Netherlands, regional PCTs have been established since 2004 [21]. The first hospital PCT was established in 1993 [22], with a strong rise in the number of hospital PCTs between 2014 and 2017. Currently, there are 34 regional PCTs and every hospital has its own hospital-based PCT [19, 20, 23].

**Fig. 1:**
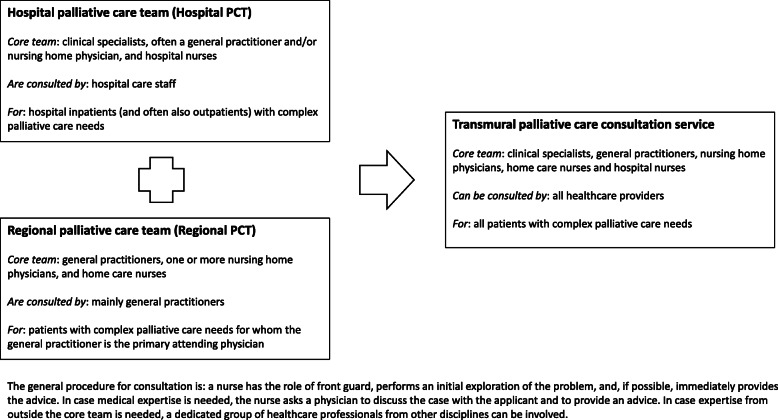
Main models of palliative care consultation services in the Netherlands

Integration of regional and hospital-based PCTs into a transmural palliative care consultation service may facilitate continuity of care for patients receiving care in different settings. However, such integration has proven to be complex and there is no evidence-based or experience-based best model available. To better understand how a transmural palliative care consultation service can be implemented, we studied the facilitators and barriers that affected the process of developing and implementing a transmural palliative care service in four palliative care networks in the southwest region of the Netherlands. The main models of palliative care consultation services in the Netherlands are presented in Fig. 1.

## Methods

### Study design and setting

In an inventory of challenges in palliative care as experienced in eight palliative care networks in the southwest region of the Netherlands, transmural collaboration was unanimously identified as the main issue. Based on this finding, networks were invited to participate in an action research program on transmural collaboration in palliative care. Within this program, four independent palliative care networks opted for the bottom-up development of a transmural palliative care consultation service within their network. Experiences in these four networks were used for this multiple case study, with researchers (ME and AvdA) closely observing the process and participating in meetings of the project teams. A multiple case study design allows for comparisons to be made across a number of cases and can serve ‘to generate in-depth, multi-faceted understanding of a complex issue in its real-life context’ [24]. The development and implementation of the transmural consultation service in four network regions were considered as four cases. An action research approach was used for data collection and data analysis. Action research is context-bound and participative, and aims at improving practice. It is a continuous learning process in which the researcher learns and also shares newly generated knowledge with those who may benefit from it [25].

In general, a regional PCT consists of general practitioners, one or more nursing home physicians, a hospice physician and home care nurses with palliative care expertise. A hospital based PCT consists of several medical specialists, often a general practitioner and/or nursing home physician, and hospital nurses with palliative care expertise. In each network a project team was formed. The size and composition of the project teams varied. Some project teams consisted mainly of palliative care experts working for either the existing regional or hospital PCT, others also included representatives from the participating organisations’ management. The total number of meetings of the project teams within a period of about 30 months varied between 5 and 18 (Table 1).

**Table 1:** Characteristics of regional palliative care networks (networks), palliative care teams and project teams

Network characteristics	A	B	C	D
Number of inhabitants in network region in 2017	415.000	460.000	380.000	425.000
Number of deceased patients in network region in 2017	3958	4286	3722	4275
Number of hospitals in network region	1	1	2	2
Number of member organisations	10	20	23	28
Geographical characteristics	A medium-sized city with surroundings	A medium-sized city with surroundings	Three rural sub- regions, with medium-sized cities as well as rural regions	Two sub-regions: a medium-sized city with surroundings and a rural region
**Palliative care team (PCT) characteristics**				
Baseline situation in 2017	One hospital PCT and one regional PCT (based in another region)	One hospital PCT and one regional PCT	Two hospital PCTs and one regional PCT	Two hospital PCTs and one regional PCT
	No earlier attempts to develop one transmural palliative care consultation service	At the start of the current program, both teams had already prepared a business plan for further integration of the hospital PCT and regional PCT	At the start of the current program, the hospital PCTs and regional PCT already shared consultants. No earlier attempts to develop one transmural palliative care consultation service	No earlier attempts to develop one transmural palliative care consultation service
Hospital PCT				
• Number of consultations in **2017**	56	33	For one hospital: unknown, for the other hospital: < 3	Unknown
Regional PCT				
• Number of consultations in **2017**	< 10^a^	11	26	47
**Project team characteristics**				
Total number of members of the project team	10	14	11	11
Members of the project team				
• Physicians				
- General practitioner	1	1	1	2
- Hospital physician	–	3	2	1
- Nursing home physician	1	2	2	2
• Nurses or nursing managers				
- Home care nurse	1	1	–	–
- Hospice nurse	1	–	–	–
- Hospital nurse	2	2	2	2
- Nursing home nurse	1	1	1	–
Others:				
• Project team coordinator	1	1	1	1
• Network coordinator	1	1	1	1
• Representative of the Netherlands Comprehensive Cancer Organisation (IKNL)	–	1	–	1
• Researcher	1	1	1	1
Number of meetings of the project team	14	18	9	5 and several meetings of sub-teams

^a^This network had no regional PCT. Healthcare providers could consult a regional PCT in a nearby region

### Data collection

The process of the development and implementation of a transmural palliative care consultation service started early 2017 and, for this analysis, ended in December 2019. We collected mostly qualitative data in all four networks, during meetings of the project teams and of the PCTs.

In concordance with our action research approach, the data collection was open and responsive to the needs of the project teams [25]. The researchers collected data during observations of meetings, informal conversations and documents. During the meetings, the researchers made minutes and field notes on processes of communication and collaboration between project team members and within PCT’s, and on the barriers and facilitators they perceived. They also took notes of individual contacts with project team members, and collected project plans and other information related to the development and implementation of the transmural palliative care consultation service. Project teams were regularly provided with feedback and the research team regularly discussed which data should be further elaborated.

The Consolidated Framework for Implementation Research (CFIR), that identifies factors that influence an intervention’s implementation, was used as a conceptual framework in the data collection and analysis [26–28]. The CFIR includes five major domains, each consisting of a number of constructs [26, 28]:

1. Intervention: the features of the intervention that is implemented. Constructs within this domain are, for example, the relative advantage of implementing the intervention versus an alternative solution, and perceived difficulties of the intervention.
2. Inner setting: the features of the implementing organisation. Constructs within this domain are, for example, the implementation climate: the degree to which stakeholders perceive the current situation as needing change, the level of priority attached to the intervention, organisational incentives, the degree to which goals are clearly communicated, and readiness for implementation.
3. Outer setting: the features of the external context or environment. This domain includes, for example, relevant external policies and incentives.
4. Characteristics of individuals involved in the implementation. This domain includes, for example, individuals’ knowledge and beliefs about the intervention and individuals’ identification with ‘their’ organisation and its goals.
5. Implementation process: the strategies or tactics used for the implementation of the intervention, such as planning, executing, reflecting, and evaluating.

### Data analysis

Data from observations, conversations, meetings and documents were analysed using the five major domains of the CFIR, to get insight into the characteristics of each case, including barriers and facilitators. For each case, we listed all factual information within the appropriate domain and underlying construct of the CFIR. Within this information, we identified elements that could potentially be considered as facilitator or barrier for developing and implementing a transmural consultation service. Emerging facilitators and barriers were further investigated in an iterative process of data collection and data analysis. Finally, for each case we had a list of facilitators and barriers within each domain of the CFIR. Subsequently, all findings were pooled together. All steps in the analytical process were initially performed by the researcher who collected the data within a case (ME or AvdA), in collaboration with the project team involved. Their initial findings were regularly discussed by the research team until consensus on the interpretation of the findings was reached.

## Results

### Characteristics of networks and PCTs

The four palliative care networks participating in this study were comparable in terms of geographical size and annual number of deaths within the network region (Table 1). The number of member care organisations varied from ten to twenty-eight. At the start of the project, each network included two or three PCTs, one regional and one or two hospital-based.

In the following account of the results of the project, we distinguish the perspectives and experiences of project team members, who had the task of developing a model for the transmural consultation service (Fig. 1), from those of the healthcare professionals working for the service. For each of the domains from the CFIR, the facilitators and barriers found are summarised in Table 2.

**Table 2:** Facilitators and barriers that affected the process of developing and implementing a transmural palliative care consultation service

Domain	Constructs	Main findings
Intervention characteristics	The relative advantage of implementing the intervention	***Facilitators****:* Healthcare professionals perceived the added value of the intervention as • improving continuity of care for patients with a limited life expectancy, regardless of the care setting; • a potential vehicle function for other activities in the field of palliative care; • promoting more transmural collaboration between palliative care experts from hospital and from primary care.
	Perceived difficulties of the intervention	***Barriers****:* • Healthcare professionals had different views on goals and activities of the transmural palliative care consultation service. • Scientific evidence for a complex intervention such as a transmural palliative care consultation service is scarce. • Where the researchers presented scientific evidence that supported (part of) the complex intervention, project teams often questioned presented findings and doubted whether these were applicable in their case.
Inner setting	The implementation climate: the level of priority attached to the intervention, organisational incentives, the degree to which goals are clearly communicated	***Facilitators****:* • Networks identified transmural collaboration as an important challenge. • Initial support from the management of involved care organisations was perceived as supportive. ***Barriers****:* • Healthcare professionals experienced limited positive stimuli from involved care organisations. • Involved care organisations sometimes had limited interest/doubts/resistance regarding the development of a transmural consultation service, because they felt there was insufficient evidence to demonstrate its benefits and cost-effectiveness. • Project teams experienced a lack of ‘best practices’ or other guidance in how to organise the service.
	Readiness for implementation	***Facilitators****:* • Networks voluntarily opted to participate in the project. ***Barriers****:* • The management of involved care organisations sometimes turned out to be reluctant when concrete efforts were required. • Registration of transmural consultations in patients’ medical files was found to be complex because of different registration systems within and outside the hospital.
Outer setting	Relevant external policies and incentives	***Facilitators****:* • Project team members considered the Netherlands Quality Framework Palliative Care and the Multidisciplinary Standards for Oncological Care in the Netherlands (SONCOS norms) for hospitals as very supportive. ***Barriers****:* • Healthcare professionals and other participants involved expressed a need for national guidance, but also wanted to adapt the intervention to the local situation. • Separate funding streams for the financing of intra- and extramural palliative care consultations made the administrative part of the transmural consultation service complex. • On top of the regular reimbursement, extra financial support was needed, but often lacking.
Characteristics of individuals involved in the implementation	Individuals’ knowledge and beliefs about the intervention	***Facilitators****:* • Palliative care experts participating in the transmural palliative care consultation service experienced collaboration and mutual exchange of information as important. ***Barriers****:* • Professionals from different care organisations having different views on palliative care, working procedures and about who was in control over the initiative made collaboration sometimes more complex. • (After implementation:) Several extra efforts being required from healthcare professionals participating in the transmural palliative care consultation service, without diminishing their regular tasks, made healthcare professionals reluctant to do, for example, extra tasks for the transmural consultation service such as attending meetings. • Different participants (healthcare professionals, coordinators, managers) asked for different motivators for the actions needed.
	Individuals’ identification with ‘their’ organisation	***Facilitators****:* • Project team members wanted to share their clinical expertise and experience within the project team and/or the transmural consultation service. ***Barriers****:* • Identification of project team members with other than their own care organisation in the palliative care network was limited. • Roles and responsibilities of project team members regarding the development of transmural procedures were not clear.
Implementation process	Planning Executing Reflecting Evaluating	***Facilitators****:* Professionals from different care organisations were dedicated and enthusiastic about the initiative. ***Barriers****:* • Professionals had difficulties to arrive at a problem definition, a concrete goal and appropriate actions. Professionals perceived limited/no guidance on how to write a project plan and how to develop and implement a transmural consultation service in the relatively complex area of palliative care.

### Intervention characteristics

In all networks, project team members agreed that the core added value of a transmural consultation service is the improvement of the continuity of palliative care for patients throughout their illness trajectory, regardless of the care setting. Continuity of palliative care can also be a challenge when patients are transferred from one care setting to another, for example for patients going home after a hospital admission, where GPs may not be adequately informed about patients’ needs, their prognosis or agreements about care and treatment. Some project team members thought that transmural collaboration can also increase the awareness of healthcare professionals in different settings of the availability of an expert consultation service and serve as a vehicle for other activities in the field of palliative care, such as education or transmural collaboration.

*In network A, during a meeting in which the project team discussed the main bottlenecks related to continuity of care in their network, they mentioned as a common problem that patients with complex palliative care needs living at home, are often hospitalised more than once in the last months of their life. One reason seems to be that hospital physicians find it difficult to actively give responsibility to the general practitioner for palliative care during this phase. Further, general practitioners and community nurses do not always know how to fulfil their role in palliative care. A transmural consultation service was expected to help solve this bottleneck in the continuity of care by advising all involved physicians on the best care for a patient and by supporting care providers in the organisation of palliative care at home.*

It was considered important that the transmural consultation service includes healthcare staff from different care settings and different disciplines, to be able to address diverse medical and nursing problems; if necessary, other disciplines should be available for problems in other domains. An actively involved hospital physician, who lobbies for support from the hospital management, turned out to be a facilitator for the development of the service. Some members of the project teams wanted to build their project plan on scientific evidence, because they thought that would help to get support from their management. However, scientific evidence for a complex intervention such as a transmural consultation service is scarce. In addition, where the researchers presented scientific evidence that supported (part of) the complex intervention, project teams often questioned presented research findings and doubted whether these findings were applicable in their case.

### Inner setting

In three networks, participants perceived the project as bottom-up initiated. In the fourth network project team members perceived the project as top-down initiated. In all project teams most participants often perceived the ownership of the initiative as unclear. Healthcare professionals participating in the project often felt insufficiently supported by their management. Before the start of the project, all networks had identified transmural collaboration as an important challenge in palliative care. They voluntarily opted to participate in the project and to develop a transmural consultation service in their region. After the start of the project, however, project teams experienced a lack of ‘best practices’ or other guidance in how to organise the service. They further felt that establishing a transmural consultation service is by definition complex, because of the involvement of different care organisations. The management of the care organisations involved in the project sometimes turned out to be reluctant when confronted with the efforts that were required for the actual development or implementation of the service.

*In network D, the manager of a transmural care organisation attended a meeting of the project team. During this meeting, the project team coordinator asked the manager about her expectations of the transmural palliative care consultation service. The manager replied that the board of the transmural care network agreed with the project on the condition that concrete results would be achieved. She added that the project team was free to determine themselves how the transmural palliative care consultation service would be organised.*

*In network A, a project plan for the transmural palliative care consultation service was submitted to the board of the palliative care network. In the project plan, the project team explained that a transmural palliative care consultation service may facilitate continuity of palliative care, but that there is limited evidence on benefits and costs. The board responded that the project plan was not sufficiently scientifically substantiated.*

They sometimes felt that there was insufficient evidence to demonstrate its benefits and cost-effectiveness. Overall, project team members reported that they received insufficient manifest positive response or rewards for their efforts and their time investments in the project from the management of the care organisation they represented, despite explicit support of the management prior to the start of the project.

A practical barrier was that registration of transmural consultations in patients’ medical files can be complex, e.g. because electronic registration systems within and outside the hospital are different and not matching.

### Outer setting

National quality frameworks for palliative care were considered very supportive by project team members. These frameworks include the ‘Netherlands Quality Framework for Palliative Care’ and quality frameworks for oncology care, such as the Multidisciplinary Standards for Oncological Care, that both promote transmural collaboration between palliative care experts from hospital and primary care.

*In network C, the network coordinator stated: “Then the Multidisciplinary standards for oncological care (SONCOS standards) came, which state that establishment of a palliative care expert team within the hospital is a requirement. Which has now been done, and what is nice in terms of initiating new developments is that, in collaboration with [name hospital] and general practitioners, we now organise palliative care consultation meetings every Wednesday, for nurses, general practitioners, but also other disciplines.”*

Healthcare professionals and other participants expressed a need for national guidance, but also wanted to adapt the intervention to the local situation. Financial reimbursement of the efforts of the transmural consultation service was mostly considered as a substantial barrier. In the Netherlands, all registered consultations outside the hospital provided by healthcare professionals from regional PCTs are financially supported from an earmarked government grant. For their coordination and other organisational issues these regional PCTs are supported by the Netherlands Comprehensive Cancer Organisation (IKNL).^1^ In contrast, hospital PCTs get no extra reimbursement per registered consultation. Reimbursement for the registered consultations, coordination and other activities of the hospital PCT is part of a total budget for all specialist medical care activities, with some hospitals being more generous towards activities regarding palliative care than others. Most, but not all hospitals participating in the project were willing to continue their financial support for the PCT after its transition into a transmural consultation service. Several physicians and nurses involved in palliative care consultation nevertheless indicated that reimbursement for their working hours for the existing hospital or regional PCT was already insufficient, with the efforts for the transmural service coming on top of this. Further, project team members indicated that besides financial support, training, physical space and time are needed to be able to start and continue a palliative care consultation service.

### Characteristics of individuals involved in the implementation

Involvement of different disciplines in project teams made the process sometimes more complex. We observed e.g. differences in professional jargon, views on palliative care, meeting habits and experience with policy making processes. In all four project teams, processes were sometimes also complicated because opinions differed about who was in control over the development and implementation of the service and about which steps needed to be taken when and by whom. Most project team members tended to identify mainly with their own care organisation. Several project team members found it difficult to collaborate with physicians or nurses from other care organisations whom they did not know. Tasks and responsibilities of the project team and project team members were often not well defined. Project team members expected that the project team coordinator would initiate the development of transmural procedures, whereas the coordinators expected initiatives from project team members, based on their clinical expertise and experience. Physicians and nurses working for the transmural palliative care consultation service were mostly very dedicated and enthusiastic, but they also felt that being a member of the project team and/or the transmural consultation service involved extra efforts: meetings of the project team and the transmural consultation service were e.g. scheduled during their regular work time, without diminishing their regular tasks. Further, working in an unfamiliar environment may be strenuous, for example for community care nurses who had to attend meetings of the service within the hospital. Healthcare professionals from different care organisations often had varying views on palliative care, working procedures and on what activities may be expected from the service. For some, the emphasis was on providing advice on care for individual patients with complex problems. Others felt that they should also organise other activities in the area of palliative care, such as education and activities to advocate palliative care. In some cases, hospital-based healthcare providers and healthcare providers working outside the hospital did not manage to develop a shared view on the activities of the transmural palliative care consultation service.

*In network B, general practitioners and hospital physicians participating in transmural meetings experienced the mutual exchange of their expertise as important. General practitioners indicated that they felt they relatively often contributed by providing information on important psychosocial and spiritual aspects of patients’ care, with hospital physicians focusing more on medical aspects and quick solutions for patients’ problems. Hospital physicians indicated that they had difficulties with time-consuming discussions of individual patients. Community care nurse consultants experienced a barrier to actively participate in transmural meetings in the hospital, due to hierarchal relationships and time pressure.*

In addition, different participants asked for different motivators for the actions needed. Physicians and nurses needed inspiring examples from other regions, and time and recognition for extra efforts for the transmural consultation service. On the other hand, coordinators and managers often felt a need for scientific evidence.

#### Implementation process

In all four networks, agreeing about the steps in the implementation process was more time consuming than expected. Project team meetings were often focused at sharing experiences of problems in transmural collaboration and it turned out to be difficult to arrive at a concrete problem definition, a concrete goal and appropriate actions for the project. Project plans for implementation of the transmural consultation service varied from global to elaborated in detail.

*In network C, the service was started after the establishment of one common telephone number in one of the two hospitals, for all palliative care consultations in the network, despite the lack of a formal implementation plan. This was mainly because this hospital strongly supported the development of a transmural consultation service. However, during the project, the two hospital PCTs and the regional PCT gradually returned to working more separately again, despite this shared telephone number.*

During the project, three out of four networks managed to develop and implement some level of a transmural palliative care consultation service (Table 3). Organisation models for the transmural consultation service varied, but a common characteristic was that a nurse has the role of front guard for consultations from both hospital and non-hospital healthcare professionals. The nurse performs an initial exploration of the problem underlying the request for an advice, and, if possible, immediately provides the advice. In case advice from a physician is needed, depending on the problem, the nurse asks a hospital or non-hospital physician consultant to discuss the case with the applicant and to provide an advice.

**Table 3:** Characteristics of the transmural palliative care consultation services

Network characteristics	A	B	C	D
Outcome in 2019	The existing hospital PCT collaborates at some level with the newly established primary care PCT.	The hospital PCT and the regional PCT collaborate in providing a transmural palliative care consultation service.	The hospital PCTs and the regional PCT collaborate in providing a transmural palliative care consultation service.	No transmural palliative care consultation service was developed.
Composition of the (transmural) PCT that provides the transmural palliative care consultation service				
• Physicians				NA
- General practitioner	3	2	4	
- Hospice physician	1	1	–	
- Hospital physician	4	6	3	
- Nursing home physician	4	2	2	
- Intellectual disability physician	1	–	–	
• Nurses				
- Home care nurse	–	–	2	
- Hospice nurse	–	2	1	
- Hospital nurse	2	2	7	
- Nursing home nurse	–	1	–	
Transmural palliative care consultation service				
• Number of consultations in **2019**	173	78	72	NA

In two networks, the service, besides providing advice for patients who present with complex problems, also organises transmural multidisciplinary meetings to discuss patient cases and training in palliative care for physicians and nurses from various healthcare settings. In the third network, the hospital PCT and the regional PCT jointly provide palliative care training sessions for non-specialised healthcare professionals. Future aim of this service is that both teams increase their collaboration to form a really integrated transmural palliative care consultation service.

## Discussion

### Summary of findings

Healthcare professionals are motivated to collaborate in a transmural palliative care consultation service but the development of such a service is a time consuming and complex process. Several facilitators and barriers play a role. The representation of multiple care organisations in the project teams made the development and implementation of a transmural palliative care consultation service complex. Healthcare professionals from different care organisations differed in perspectives on palliative care and working procedures. When developing the transmural consultation service, they mostly found ways to deal with these differences. Support of the management of several care organisations, which is needed to involve sufficient palliative care experts, was sometimes lacking. Those involved in the development of the transmural consultation service felt that they were insufficiently supported for their efforts and time investments.

### Healthcare professionals’ perspectives on palliative care and transmural collaboration

Healthcare professionals working for the consultation service considered national quality frameworks for palliative care [29, 30] as facilitating and supportive to substantiate the importance of the initiative. However, it turned out to be difficult to find a shared view on concrete goals and activities of the service, whereas having shared goals and visions is considered to be an important dimension of transmural collaboration [31, 32].

One of the barriers was that healthcare professionals from hospital and primary care working for the consultation service experienced difficulties in transmural collaboration, due to differences in their respective input in meetings, differences in focus and in time spent per case, and differences in professional language use. Also healthcare professionals sometimes felt uncomfortable due to differences between the hierarchical structure in the hospital environment and primary care. These findings are in line with other studies [33, 34]. In a review on advance care planning (ACP) for patients with cancer, Kuusisto et al. found differences between hospital physicians and general practitioners in their opinion on the appropriate timing of the start of the palliative phase, and in how to continue conversations about preferences for care throughout the illness process [33]. In a descriptive qualitative study on healthcare providers’ views on the transition between hospital and primary care for patients in the palliative phase, Flierman et al. found similar differences in views on when and how patients should be informed about their limited life expectancy between hospital and primary care professionals [34]. Therefore, whereas transmural collaboration is essential, it also brings challenges. It took relatively much effort to make healthcare professionals involved agree about the bottlenecks in palliative care in their network and about the added value of transmural collaboration. Views on palliative care differed between healthcare professionals from different organisations and also between healthcare professionals within organisations. It is known from other studies that especially nurses find it difficult to describe their role and responsibilities in palliative care [35]. Our findings show that actually all healthcare professionals find it difficult to describe their role and responsibilities in transmural collaboration in palliative care. For a transmural consultation service, more shared views on palliative care and the roles and responsibilities of involved healthcare professionals from hospital and regional PCTs are needed.

Our findings show that in the one network in which participants perceived the project as top-down initiated no transmural consultation service was developed. The baseline situation in the networks thus seemed a predictor of success and may be an indicator for how to coordinate or manage innovations in this area.

### Organisation of a transmural palliative care consultation service

Support of the management of healthcare organisations was found to be a facilitator for the initiative, especially support of the local hospital(s) involved. However, throughout the project, care organisations sometimes expressed doubts about the intended effects of a transmural consultation service. The lack of evidence regarding the effectiveness of transmural consultation services contributed to these doubts. In addition, the care organisations that were involved in the project are participants of a network that has been set up to promote transmural collaboration in palliative care, but in which collaboration and responsibilities are not always formalised [31]. Our findings show that the unclear ownership of the initiative was a barrier for transmural collaboration. We found that the management of different healthcare organisations sometimes had different ideas about the organisation of palliative care and about their own role in transmural collaboration. With unclear ownership of the initiative it turned out to be difficult to get everyone on the same page.

A difference in focus between professionals and managers was also identified as a barrier for the organisation of a transmural consultation service. Healthcare professionals who were already working for either a hospital or a regional PCT focused on the best palliative care for the individual patient whereas the management of healthcare organisations focused on evidence-based benefits and costs. We found that different stakeholders asked for different motivators, which slowed down the process. Other studies also showed that, although perceived as important, transmural collaboration between healthcare professionals from different care organisations is complex [36]. In a systematic review on values of integrated care, it was found that healthcare professionals and the management of healthcare organisations associate different values with transmural collaboration. The management attached more importance to general values such as ‘cost-effectiveness’ and ‘evidence-based practice’, whereas healthcare professionals attached more importance to values that are specifically relevant for collaboration, such as ‘collaborative attitude’, ‘co-ordination’ and ‘co-production’ [37].

Although healthcare professionals perceived the national quality frameworks as supportive, because they emphasise the importance of transmural collaboration for good-quality palliative care, these frameworks offer no practical guidance regarding the best way to organise transmural collaboration. Further, reimbursement systems for hospital and non-hospital consultations and other activities from the hospital and regional PCT vary greatly in the Netherlands [38]. In addition, hospital and other care organisations often have different registration systems, and privacy legislation makes it difficult to exchange information between healthcare professionals from different care organisations [39].

Another barrier was that several healthcare professionals felt that activities for the transmural palliative care consultation service were not perceived, by others or by themselves, as a serious part of their regular work. Healthcare professionals were especially reluctant towards taking responsibility for organisational tasks for the transmural consultation service. Reasons for this, as was also found in other studies, were time pressure in their daily activities due to increased efficiency requirements [40–42], but also that healthcare professionals tend to focus on what they consider medical or nursing aspects, and distance themselves from what is considered care coordination or organisational tasks not directly related to an individual patient [41, 43, 44]. Based on our findings, it can be questioned to what extent it can be expected from healthcare professionals to carry out organisational tasks in the development and implementation of a transmural consultation service, where it is unclear if these tasks are part of their regular work and where their efforts are hardly acknowledged. Following the results of our study, healthcare professionals need more guidance on and support in organisational issues regarding transmural collaboration in palliative care. Measuring the impact of transmural palliative care at several levels has been shown to be complex [32, 45]. However, more scientific evidence of what a transmural consultation service contributes to continuity of palliative care in terms of benefits and ‘cost-effectiveness’ would be helpful in getting more management support.

## Strengths and limitations

A strength of this multiple case study is that it provides valuable insight into the development and implementation of a transmural palliative care consultation service in four palliative care networks. Two researchers, each in other networks, followed the process in project teams during 2–3 years and were able to collect in-depth information about the process in the four networks. A limitation is that the CFIR may not be entirely appropriate for research on transmural collaboration in palliative care. However, the CFIR is based on relevant implementation theories in various disciplines [26] and offered a clear structure for data collection and analysis. Because of the similarities and differences between the networks found during the study, it is expected that the findings are applicable to similar contexts in other parts of the Netherlands and Europe.

## Conclusion

In conclusion, healthcare professionals were motivated to collaborate in a transmural palliative care consultation service, because they believe it can contribute to high-quality palliative care. Facilitators for developing the service were support of the management of several healthcare organisations, and national quality frameworks for palliative care. However, more shared views on goals and activities of a transmural palliative care consultation service are needed, as well as more guidance on how to organise such a service in the complex area of palliative care. A clear and comprehensive financing system is another prerequisite. Healthcare organisations should provide healthcare professionals involved with consistent and explicit support and reward them for their efforts in such an initiative. Finally, more research is needed on benefits and ‘cost-effectiveness’ of different models of integrated transmural palliative care consultation services.

## Data Availability

The datasets generated and analysed during this study are stored at the Erasmus MC, University Medical Center Rotterdam, Department of Public Health, Rotterdam, the Netherlands. The data are not publicly available given the confidential nature.

## Abbreviations

CCMO: Central Committee on Research Involving Human Subjects (in Dutch: Centrale Commissie Mensgebonden Onderzoek)
CFIR: Consolidated Framework for Implementation Research
MD: Doctor of Medicine
PCT: Palliative Care Team
SONCOS: Dutch Federation of Oncological Societies (in Dutch: Stichting Oncologische Samenwerking (SONCOS))

## Footnotes

1 Nowadays, the regional PCTs are supported by Fibula, an organisation that focuses on the organisation of palliative care and stimulates partnerships.
